# Reduced patient anxiety as a result of radiation therapist‐led psychosocial support: a systematic review

**DOI:** 10.1002/jmrs.208

**Published:** 2017-02-03

**Authors:** Kelly Elsner, Diana Naehrig, Georgia K. B. Halkett, Haryana M. Dhillon

**Affiliations:** ^1^ Central Clinical School, Sydney Medical School University of Sydney Sydney Australia; ^2^ School of Psychology, Faculty of Science University of Sydney Sydney Australia; ^3^ School of Nursing, Midwifery and Paramedicine, Faculty of Health Sciences Curtin University Perth Western Australia Australia; ^4^ Centre for Medical Psychology and Evidence‐based Decision‐making University of Sydney Sydney Australia

**Keywords:** Communication, patient anxiety, patient care, psychosocial care, radiation therapist, systematic review

## Abstract

Up to 49% of patients attending radiation therapy appointments may experience anxiety and distress. Anxiety is heightened during the first few visits to radiation oncology. Radiation therapists (RT) are the only health professionals in direct daily contact with patients during treatment, placing them in a unique position to explore patients’ psychosocial needs. This review aims to synthesise literature regarding the effect of RT‐led psychosocial support on patient anxiety. In May 2015, we searched the following electronic databases: Medline, PsycINFO, Embase, CINAHL, PubMed and Cochrane library. Radiation therapy‐specific journals were hand‐searched, and reference lists of identified studies searched. This review complies with Preferred Reporting Items for Systematic Reviews and Meta‐analyses (PRISMA) guidelines. The search identified 263 articles, of which 251 were excluded based on non‐English language, duplicate article or relevance. A total of 12 articles involving 1363 patients were included and categorised into three broad themes: ‘Patient Perspectives’ 3 articles, ‘Patient Information and Education’ 5 articles and ‘Screening and Needs Assessment’ 4 articles. Two publications referred to the same sample and data. Quality ratings were mixed, with one study rated ‘high’ quality, seven ‘moderate’ and four ‘low’. Methodological weaknesses were identified in relation to workflow, sample size and responder bias. RTs have a role in psychosocial support through increased communication and information sharing, which can benefit both patients and staff. RT‐led practices such as relationship building, patient education sessions and screening and needs assessments are feasible and can reduce anxiety.

## Introduction

It is widely documented that up to 49% of patients attending radiation therapy appointments may experience anxiety and distress.^1^, ^2^ Anxiety is heightened during the first few visits to radiation oncology, particularly prior to starting treatment.^2^, ^3^, ^4^, ^5^ During these visits, patients meet a variety of health care professionals (HCPs), including radiation oncologists (ROs), radiation therapists (RTs) and radiation oncology nurses (RONs). RTs’ primary roles are patient care, radiation planning and treatment delivery. Their role incorporates patient education, including explanation and co‐ordination of procedures and appointments, and providing advice regarding personal care during treatment.^6^ In fulfilling these roles, RTs need to spend time with patients to ensure their information needs are met and that they are willing to proceed with treatment.^3^, ^7^ Consequently, RTs have a role in providing psychosocial support to patients, but this role is not well defined.

RTs are the only HCPs in direct daily contact with patients during treatment, placing them in a unique position to explore patients’ psychosocial needs.^1^, ^8^ Up to one third of patients treated with radiation therapy have been identified as having unmet psychosocial needs with respect to information and communication, emotional and spiritual support, management of physical symptoms and involvement of family and friends.^9^ These unmet needs can result in refusal to undergo radiation therapy, treatment delays, reduced compliance, low adherence to medical advice, decreased quality of life, decreased satisfaction with services and increased resource use.^5^, ^10^ It may be possible to improve the quality of care for patients treated with radiation therapy by addressing their unmet psychosocial needs; however, there have been few studies in this area and no systematic reviews. This systematic review aims to synthesise literature regarding the effect of RT‐led psychosocial support on patient anxiety.

## Methods

This review complies with Preferred Reporting Items for Systematic Reviews and Meta‐analyses (PRISMA) guidelines.^11^

### Search strategy

Qualitative and quantitative studies were identified across electronic databases: Medline, PsycINFO, Embase, CINAHL, PubMed and Cochrane library. The search conducted in May 2015 included the following terms: (radiation therapist, radiotherapist, radiographer or technologist) and (psychosocial, supportive, psychol*, rapport, relationship, communication, psychoeducation, social support, patient education, patient satisfaction or health communication) and (patient) and (anxiety, depression, stress, distress or coping). Hand‐searched journals included *Journal of Medical Radiation Sciences*,* The Radiographer*,* Radiation Therapist* and *Journal of Radiotherapy in Practice*. Reference lists of identified studies were also searched.

### Screening

**Figure 1:**
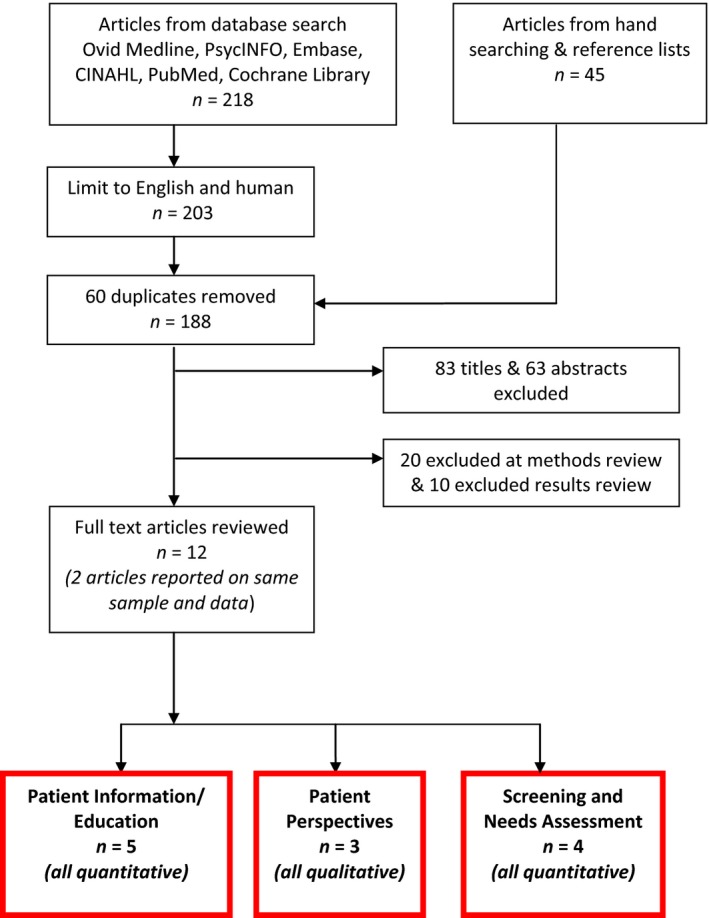
PRISMA flow diagram of search results.

Initial search results were checked for duplicates (see Fig. 1). Titles and abstracts were independently screened by authors (K.E., H.M.D.) and studies were excluded according to pre‐determined PICO criteria (see Table 1). Discrepancies were resolved by discussion. Remaining studies were subjected to blinded examination of methodology to assess eligibility.

**Table 1:** PICO definitions of inclusion criteria

PICO	Inclusion criteria
Population	Radiation therapists or radiation therapy patients receiving external beam treatment
Intervention	Radiation therapist led
Comparison	With or without control group
Outcomes	Patient‐related: anxiety, depression, distress, quality of life, self‐reported side effects and symptoms, satisfaction, adherence to treatment, unplanned admissions; Radiation therapist‐related: perceptions, confidence, communication or feasibility of intervention.
Study type	Any

### Data extraction and analysis

Author, K.E., extracted the following data: type and aim, participants, timing and measurement, intensity and feasibility. PRISMA guidelines were used to identify quality criteria and risk of bias, without knowledge of study results^11^ (see Table 2). Subsequently, a quality rating of ‘high’, ‘moderate’ or ‘low’ was assigned. No article was excluded on quality alone, and all authors reached consensus on quality ratings via discussion. Full text copies of potentially relevant articles were obtained, and results and reported outcomes were extracted. A meta‐analysis was not feasible, due to the diversity of interventions, measures and outcomes, thus a qualitative synthesis is presented.

**Table 2:** Quality rating criteria for included studies

Type	Number	Criteria
Outline of quality rating criteria		
Quant	1	Intervention details: type, aim, timing, measurement, intensity, feasibility
Quant	2	Risk of bias assessed Concealed – Blind or double blind Method of allocation including sequence generation and concealment from recruiters
Quant	3	Control group in study design
Quant	4	Measurement tools validated Validity, reliability addressed Generalisability
Qual	5	Research credible? (data fitting to views of participants) Research dependable/reliable? (logical, traceable, clearly documented) Research confirmable/objective? (analysis grounded in data, researchers bias stated and explored)

First author and year	Study type	Criteria number					Overall quality rating
		1	2	3	4	5	
Included studies rated according to criteria							
Halkett et al^19^ (2013)	Quant	H	H	H	H	N/A	H
Dong et al^15^ (2014)	Quant	H	N/A	N/A	M	N/A	M
Braeken et al^20^ (2011)	Quant	H	M	H	M	N/A	M
Clover et al^4^ (2011)	Quant	H	N/A	N/A	M	N/A	M
Oultram et al^12^ (2012)	Quant	H	N/A	N/A	M	N/A	M
Halkett et al^17^ (2012)	Quant	M	N/A	N/A	M	N/A	M
Mitchell and Symonds^21^ (2012)	Quant	M	N/A	N/A	M	N/A	M
Canil et al^16^ (2012)	Quant	L	N/A	N/A	L	N/A	L
Miller^18^ (2008)	Quant	L	N/A	N/A	L	N/A	L
Halkett and Kristjanson^3^ (2007)	Qual	N/A	N/A	N/A	N/A	M	M
Egestad^13^ (2013)	Qual	N/A	N/A	N/A	N/A	L	L
Egestad^14^ (2013)	Qual	N/A	N/A	N/A	N/A	L	L

H, high; M, moderate; L, low; N/A, not applicable; Quant, quantitative; Qual, qualitative.

## Results

The search identified 263 articles, of which 251 were excluded based on non‐English language, duplicate or relevance during title, abstract and methods review (see Fig. 1). In total, 12 articles, involving 1363 patients, were included. Most were conducted at single centres and included patients 18 years or older. The most common reasons for patient exclusion were too unwell, identified cognitive deficits or unable to communicate fluently in the nominated language. The 12 studies were classified into 3 categories according to approach or intervention type: ‘Patient Information and Education’ 5 studies, ‘Patient Perspectives’ 3 studies or ‘Screening and Needs Assessment’ 4 studies. Results of publications using the same sample and data were reported together, this included articles by Clover et al. and Oultram et al.,^4^, ^12^ and combining Egestad.^13^, ^14^ Quality assessment and summary results are presented below and in Table 3.

**Table 3:** Summary of included studies

Author and year	Type	Target cancer diagnosis	Category	No. of patients	No. of RTs	RT training	Results
Halkett et al.^19^ (2013)	RCT	Breast	PIE	122	10	2 mandatory workshops: (1) Preparing patients for radiation therapy (2) Eliciting and responding to emotional cues	At pre‐planning time point, significant results for intervention versus control: anxiety reduced by 0.15 points, knowledge (planning) increased by 3.5 points, knowledge (treatment) increased by 5.3 points, radiation therapy‐related concerns reduced by 0.9 points.
Dong et al.^15^ (2014)	Cross‐sectional	Mixed	PIE	56	10	N/A	RTs scored high on ‘MPCC information’ (explaining radiation therapy procedures, skin care, side effects) RTs scored low on ‘MPCC feelings’ (inquiring about patient feelings/fears/anxieties, understanding of radiation therapy) Post‐consultation decrease in STAI scores (range): baseline 10.98 (6–24), post‐consultation 9.6 (6–17)
Braeken et al.^20^ (2011)	RCT	Mixed	SNA	568	7	1‐h session – use and interpretation of SIPP conducted by the researcher and 2 social workers	SIPP feasible and valued by most patients and some RTs Patient perspectives: 67.5% agreed discussing SIPP with RTs was important; 47.4% rated discussions as pleasant; usefulness of discussing physical, psychosocial and sexual issues with RTs were 56%, 39.3% and 9.3% respectively. RT 7‐month versus 13‐month FU SIPP usefulness for ‘quality of consult’ – 33.3%, 16.7% and 50.1% versus 66.7%, 0%, 33.3% negative, moderate and positive respectively RTs were negative towards changing communication styles, SIPP usefulness in referring patients to psychosocial care and feasibility of discussing psychosocial issues RTs reported increased patient communication and knowledge of patient issues through screening processes RT motivation positively correlated with ‘usefulness’ of screening processes
Clover et al.^4^ (2011) and Oultram et al.^12^ (2012)	Cohort	Head & neck or Brain	SNA	105	35	N/A	At CT‐Sim: RTs identified 27% of patient self‐reported cases of anxiety and 90% of non‐anxious cases, provided verbal reassurance alone to three patients and three patients had their mask removed At Fraction 1: RTs identified 50% of patient self‐reported cases of anxiety and 57% of non‐anxious cases; provided verbal reassurance alone to three patients, three patients had their mask removed (one refused further treatment), one patient received verbal reassurance and mask removal (two of these patients were unable to complete treatment that day). Authors concluded that patients may have under‐rated anxiety, while RTs may have over‐rated anxiety
Halkett et al.^17^ (2012)	Pre–post feasibility	Breast	PIE	13	4	2 mandatory workshops: (1) Preparing patients for radiotherapy (2) Eliciting and responding to emotional cues	HADS scores decreased from baseline to T1 and T2: Baseline mean = 13.6 (SD = 8.03, range = 2–22); T1 mean = 6.4 (SD = 4.9, range = 2–19); T2 mean = 7.0 (SD = 7.5, range = 0–20) Mean scores for ‘concerns about radiotherapy’ dropped from baseline T1, mean = 4.4 (SD = 2.45), to T2 (post‐planning intervention), mean = 2.50 (SD = 1.64) respectively ‘Knowledge of radiotherapy’ scores increased from T1 to T2 and T3 Patients reported the intervention was beneficial in preparing for treatment RTs were positive about delivering intervention and the perceived benefit to patients The intervention was feasible and acceptable Time, staffing and space were identified as barriers in delivering intervention. Time and staffing issues were remedied
Mitchell and Symonds^21^ (2012)	Cohort	Mixed	SNA	379	30	Optional 1‐h session in use of screening tool. Communications training also available. Less than 25% of clinicians attended	RTs report screening ‘useful’, ‘not useful’ or ‘unsure’ in 43%, 21.5% and 35.4% of assessments respectively Significant positive correlation between RTs rating screening as ‘useful’ and rating any of the following: the ‘screening tool as practical’, the ‘RT having low confidence’ or ‘assessing a patient with high anxiety’ Favourable perception of screening was significantly correlated with both completion of screening tool training and improved detection of psychological issues RTs reported increased patient communication and knowledge of patient psychological issues using screening
Canil et al.^16^ (2012)	Cross‐sectional	Mixed	PIE	24	N/A	N/A	Anxiety STAI‐S pre‐ and post‐test median scores were 2.00 and 1.46 respectively (*P* < 0.001). No change = 1 patient, increased anxiety = 3 patients Self‐efficacy CBI‐B pre‐ and post‐test median scores were 6.96 and 7.82 respectively (*P* < 0.001). No change = 3 patients 16 of 23 attendees reported reduced concerns Many reported reduced feelings of isolation
Miller^18^ (2008)	Cross‐sectional	Mixed	PIE	50	N/A	N/A	Post‐intervention, patients reported: feeling more confident and less anxious about treatment; meeting other patients helped decrease feelings of isolation; reassurance was gained through staff openness and friendliness Components rated most valuable were demonstration of the treatment machine 66% and informal one‐on‐one chat 34% with RT staff
Halkett and Kristjanson^3^ (2007)	Qualitative interview	Breast	PP	34	N/A	N/A	Patients perceive RTs as not only technical professionals but also information and supportive care givers Main theme: the importance of the patient achieving emotional comfort Emotional comfort is achieved by forming relationships with RTs and gaining information. Achieving emotional comfort can decrease anxiety and enables the patient to feel more relaxed, a sense of belonging and confident in the treatment and RTs skills Developing a relationship with the same RTs daily was perceived to reduce anxiety, improve continuity of information and treatment accuracy
Egestad^13^, ^14^ (2013)	Qualitative interview	Head & neck	PP	12	N/A	N/A	Main themes: emotional vulnerability, need to be treated as a unique person Sub‐themes: to be understood, emotional support, to feel safe, to form relationships, politeness and communication Patients valued effective communication, being treated as an individual, care/empathy and acknowledgement RTs who initiated relationships, spent time with patients and provided information helped decrease patient vulnerability, anxiety and loneliness Familiar RTs who provide information and build a relationship with the patient can reduce patients’ loneliness, existential anxiety and uncertainty Perceived RT incompetence can increase patient insecurities and anxiety

N/A, not applicable; NK, not known; SNA, screening and needs assessment; PIE, patient information/education; PP, patient perspectives; MPCC, measure of patient centre communication; STAI, State‐Trait Anxiety Inventory; ASR, authentic self‐representation; SIPP, Screening Inventory of Psychosocial Problems; FU, follow‐up; CT‐Sim, computed tomography simulation.

### Quality assessment

One study was rated ‘high’ quality, seven ‘moderate’ and four ‘low’. Methodological weaknesses were identified in relation to workflow, sample size and responder bias.

Workflow and sequencing of interventions and measurements may have impacted results of three studies. In these studies, patient self‐report measures were completed not only after the intervention, but also after the patients’ first treatment session, consequently, it is impossible to determine the effect of intervention alone on anxiety.^15^, ^16^, ^17^

Sample sizes were small, with four of eight quantitative studies recruiting 56 patients or less.^15^, ^16^, ^17^, ^18^ Such samples are insufficiently powered to detect small but meaningful effect sizes. Furthermore, only two studies incorporated control groups to enable assessment of intervention effect.^19^, ^20^

Responder bias may have inflated the effect of group education sessions on anxiety, as session attendance was voluntary and studies did not collect data from non‐attendees.^16^, ^18^ Canil et al.^16^ reported a skewed population including more non‐immigrants, higher socioeconomic status and English as a primary language.

### Patient information and education

All studies in this category reported decreased patient anxiety. Two studies reported results of group patient information and education sessions,^16^, ^18^ while three studies investigated one‐to‐one RT‐led education/information sessions.^15^, ^17^, ^19^ Canil et al.^16^ assessed the impact of group sessions (*n* = 24) and detected a significant decrease in anxiety (*P* < 0.001) from baseline to post‐intervention. In a cross‐sectional study completed after a group session, Miller reported that 47 (94%) patients felt more confident and less anxious.^18^ Dong et al.^15^ assessed patient centredness of one‐to‐one pre‐treatment sessions (*n* = 56) and reported a post‐consultation decrease in anxiety of 1.2 points. Halkett et al.^17^ also demonstrated one‐to‐one psycho‐educational interventions reduced anxiety (*n* = 13) from baseline (post‐radiation oncologist consultation) to radiation therapy planning and first treatment. In a pilot randomised control trial (RCT) (*n* = 122), Halkett et al.^19^ showed a greater reduction in anxiety between baseline and post‐radiation planning in the intervention group compared to usual care.

Both group and individual education/information sessions are effective in reducing patient anxiety, reducing fear of the unknown and feelings of loneliness. An increase in self‐efficacy, knowledge of radiation therapy and preparedness for treatment were also reported.^16^, ^17^, ^18^, ^19^ However, no direct comparison of individual versus group approach was found in the literature.

### Patient perspectives

Halkett et al. and Egestad^3^, ^13^, ^14^ reported congruent themes suggesting RT actions and behaviours can reduce patient anxiety. Egestad^13^, ^14^reported reduced anxiety to be associated with effective communication, being treated as an individual, active care, empathy and acknowledgement. Patient anxiety was further reduced by RTs who initiated relationships, spent time with patients and provided information.^13^, ^14^ Similarly, Halkett et al.^3^ reported that patients gained emotional comfort, a sense of belonging and increased confidence in RTs by forming relationships and receiving information. Both authors reported that seeing the same RTs daily reduced anxiety and influenced perceptions of continuity of information and care, accurate treatment delivery, safety and RT competence.^3^, ^13^, ^14^ Patients perceived RTs to be competent if they performed their technical duties quickly and confidently, were able to answer questions, recognised and managed side effects and explained unexpected events (e.g. machine breakdowns). Egestad highlighted that adverse side effects can occur, or be poorly managed, due to lack of information sharing and lack of relationship building.^14^

These studies indicate that RT–patient relationships, communication and continuity of care are important aspects of health care that reduce patient anxiety.

### Screening and needs assessment

Results in this category varied. Braeken et al.^20^ concluded that use of the Screening Inventory of Psychosocial Problems (SIPP) screening tool was feasible, with the majority of patients and RTs agreeing that screening discussions were important and pleasant. ‘Physical’ and ‘emotional’ needs were rated as acceptable to explore with screening, but ‘sexual’ issues were not. In the context of individual patient screening processes, RTs rated the SIPP highly as an ‘invitation to discuss’ and provide ‘better insight into patients’ psychosocial well‐being’. However, global assessment of the usefulness of the SIPP varied across information items and time points. At 7 months post‐study commencement, RTs highly rated SIPP as useful to ‘contribute to discussion’, ‘quality of consult’ and ‘contribution to psychosocial discussions’, but these aspects were rated poorly at 13 months.^20^ Mitchell and Symonds reported that 43% of RTs rated screening with the ‘distress and emotion thermometers’ as useful. The screening process was found to be most useful when RTs were uncertain of the presence of anxiety or when anxiety was clearly high. Mitchell and Symonds also noted that RT motivation, use of screening and detection of psychosocial issues all increased if RTs rated the screening tool as practical and relevant.^21^ Clover et al. and Oultram et al.^4^, ^12^ found slight agreement between anxiety reported by patients compared with RTs. Of those patients self‐reporting anxiety, RTs correctly identified 27% of cases of anxiety at radiation planning and 50% of cases at first treatment.

These studies indicate that RT‐led ‘screening and needs assessment’ is feasible, improves communication with patients and increases RT knowledge of patient issues.^12^, ^20^, ^21^

### Psychosocial referrals

Braeken et al. monitored psychosocial referrals made at one‐to‐one sessions between the patient and their assigned RT utilising the SIPP. During these sessions, conducted prior to commencing treatment, 33 referrals were recorded. Of patients referred, 31 demonstrated sub‐clinical or clinical psychosocial symptoms. Twenty‐one referrals were accepted, indicating an appropriate time point to offer psychosocial referrals. During sessions conducted at completion of the treatment course, nine patients, all of whom experienced clinical psychosocial symptoms, were offered and accepted psychosocial referrals.^20^

### Time to deliver screening processes and interventions

Time may be a barrier to implementing new processes. Mitchell and Symonds and Braeken et al. reported average RT–patient screening discussion times of 3 and 5.3 min respectively.^20^, ^21^ Dong et al.^15^ recorded a wide range of times, 3.36–16.17 min, in pre‐treatment education sessions during which some anxiety is addressed, suggesting variability between sessions. Halkett et al.^19^ monitored the quality of pre‐planning and pre‐treatment education consultations, hence these longer session times (mean = 24.9 min) may be more representative of time required to deliver a meaningful intervention.

### RT training

Four of 10 studies provided training to RTs prior to study commencement. Mitchell and Symonds and Braeken et al.^20^, ^21^ provided 1 h training sessions specific to the use of screening tools being tested and recognition of emotional issues. Halkett et al.^17^, ^19^ provided mandatory training consisting of two 4‐h workshops for RTs delivering the intervention. Mitchell and Symonds reported that less than 25% of participants completed training and speculated that lack of protected time to attend training was a contributing factor.^21^ Both Mitchell and Symonds and Braeken et al.^20^, ^21^ concluded that their results may have been negatively impacted by insufficient training and recommended further communication skills training (CST). Halkett et al.'s RT training workshops focused on content and delivery of radiation therapy‐specific information to patients and CST, specifically ‘eliciting and responding to emotional cues’. Real‐time feedback, ongoing mentoring and support were provided to RTs during study intervention delivery.^17^, ^19^ Oultram et al. and Dong et al.^12^, ^15^ also recommended CST to improve detection and management of patient issues including anxiety, claustrophobia, coping and side effects. Clover et al. and Oultram et al.^4^, ^12^ reported that RTs over‐estimated anxiety compared to patient self‐report, and suggested training may improve accurate detection.

### Implementation recommendations

‘Information/education’ and ‘screening and needs assessment’ interventions are feasible and improve patient outcomes.^15^, ^16^, ^17^, ^18^, ^19^, ^20^ However, they must be implemented strategically due to perceived negative impact on staffing requirements, appointment schedules and resources, for example private rooms.^5^, ^21^ Mitchell and Symonds recommended engaging motivated and non‐motivated RTs in the development process, providing training, ongoing support/mentoring and meaningful feedback and developing clear action plans.^21^ Implications are that management and frontline RTs work together to provide infrastructure to enable interventions and overcome identified barriers to achieve improved patient care and outcomes, specifically reduced anxiety.

## Discussion

This systematic review identified a small number of publications focused on RT‐led psychosocial practices including detection, assessment or management of patient anxiety. All recognised the need to address psychosocial issues and indicate that RTs can positively impact on patient experiences of radiation therapy. Specifically, RT–patient interactions can reduce patient anxiety through effective communication, forming relationships, acknowledging patients as individuals and provision of education/information. Patient anxiety could be further reduced by exploring the RT role, application of screening and needs assessments and training in both communication skills and detection and management of emotional distress.

The increasing prevalence and burden of emotional morbidity related to cancer diagnoses and survival are widely recognised. This has resulted in the development of ‘Clinical Practice Guidelines for the Psychosocial Care of Adults With Cancer’, which provide awareness and practical information to HCPs to improve the management of psychosocial issues for patients and carers.^22^ Turner et al.^23^ highlighted that most HCPs have minimal training and knowledge in this area. In fact, it has been reported that RTs are not confident discussing psychosocial issues.^24^ However, all HCPs working with cancer patients need to adhere to these guidelines in clinical practice to enable early detection of psychosocial issues, empathetic management and effective referrals to specialised care.^23^

Radiation therapy provokes high anxiety, with patients reporting fear of radiation and that being in an oncology department reminds them of their life‐threatening condition.^13^, ^14^, ^18^ RTs prepare patients for the procedure through education and information before the start of treatment. Adequate preparation has been shown to reduce patient anxiety as well as reduce recovery time and complication rates in aversive and invasive medical procedures.^25^ Furthermore, RTs interact with patients daily, and throughout treatment are able to tailor information to suit individual patient's changing needs and to involve patient's in their own care, for example, by encouraging them to ask questions.^23^, ^26^ The RT–patient rapport also enables RTs to consider whether to involve families and carers in education/information sessions which may improve the overall patient experience and potentially reduce patient and family anxiety.^5^ In summation, the RT–patient relationship is unique and valued by RTs and patients.

Confusion regarding the ‘radiation therapist’ role may contribute to a lack of patient satisfaction, information provision and psychosocial support. The role is defined by RTs and patients as encompassing technical, information and supportive care components.^6^ While the RT role will vary across departments, clear definitions and expectations could focus RT interactions and increase patient satisfaction, while ensuring patient needs are met. Braeken et al. reported that RTs were less positive about asking questions regarding patient psychosocial well‐being and patients reported that psychosocial and sexual issues were not discussed.^20^ Similarly, Dong et al. reported that in one‐to‐one education sessions RTs scored poorly when exploring patients’ feelings, fears and anxiety and understanding of radiation therapy. Interestingly, Dong et al. showed a significant positive correlation between patient‐centred communication and authentic self‐representation; thus, when more interest was shown, the patients represented themselves more honestly, expressed concerns and asked questions.^15^ This is important in the context presented by Egestad, where four of five masked head and neck patients with claustrophobia ‘forced themselves’ through radiation sessions without disclosing their fears.^13^, ^14^ It is possible, that RTs do not ask about patient psychosocial issues as they do not believe it is their role, know how to elicit information or manage concerns. This was raised by oncologists, surgeons and nurses who worried that screening a patient for psychosocial issues is not advantageous if the HCP is then unable to manage the issues disclosed due to lack of time, training, referral pathways and specialised services.^27^

RT training in the areas of communication skills (CST) and emotional well‐being could enhance the patient experience.^3^, ^7^, ^12^, ^15^, ^20^, ^21^, ^28^ Psychosocial care guidelines state that HCPs need an understanding of common conditions, such as anxiety and depression, and an awareness of effective treatments to enable detection and discussion of such issues with patients.^23^ This is supported by Mitchell and Symonds who reported RTs and chemotherapy nurses trained in use of screening tools were more satisfied with screening processes and more motivated to screen patients, discuss issues and educate patients.^21^ Braeken et al., who reported low training compliance, stated RTs did not rate psychosocial discussions as important, and RTs did not change communication styles when using the SIPP, a tool designed to explore psychosocial issues.^20^ Fallowfield et al. support these observations stating that professional experience alone does not resolve poor practitioner–patient communication, but CST can improve skills. In a study of 160 oncologists, those who completed CST showed significantly greater expressions of empathy, use of focused questions and appropriate responses to patient cues in consultations after training. Oncologists reported the training to be interesting and highly relevant to clinical practice.^10^ Similarly, in a study by Halkett et al., 60 RTs who participated in two communication skill workshops rated strong satisfaction with all aspects of the training including relevance to daily practice, increased confidence and acquisition of new skills. However, to ensure effective learning, small group sessions with opportunities to practice skills and receive feedback are essential.^5^ Furthermore, to ensure translation of learned skills into the clinical environment, clinical supervision/mentoring and feedback are recommended.^27^, ^29^ Training in emotional distress and CST, including ongoing support for RTs, could lead to improved patient‐centred care, recognition and management of patient issues and use of screening processes.

The value of the RT–patient relationship may be enhanced by using screening and assessment tools. Evidence suggests that screening tools are more successful in detecting psychosocial issues than relying on clinical judgement alone.^27^ Screening tools may facilitate triaging by RTs which could reduce burden on limited psycho‐oncology resources and provide timely patient support.^4^, ^27^ Clover et al.^4^ proposed a two‐tiered screening and intervention system, with RTs screening for anxiety and managing patients exhibiting low anxiety through skilled communication. Patients with moderate to high anxiety or psychological issues would be referred for specialised care. Turner has actioned this innovation in ‘PROMPT’, a RCT with a three‐tiered system.^29^, ^30^ Additionally, referral pathways must be clear and accessible to RTs,^7^ as various patient‐reported needs, including physical, sexual, financial and spiritual, may be better provided by multidisciplinary team members such as the radiation oncologist, nurse, social worker, counsellor, nutritionist or other.

This systematic review has some limitations. A systematic process was followed to identify relevant publications; however, it is possible that articles may have been missed or were published after the search was conducted. Researcher bias is a conceivable limitation, although this was minimised by involvement of and discussions among all authors.

## Conclusion

Evidence suggests that RTs have a role in psychosocial support through increased communication and information sharing that can benefit both patients and RTs. RT‐led practices such as education and information sessions, screening and needs assessments and relationship building are feasible and promising as moderators of anxiety and warrant further investigation using more rigorous evaluation methods. Future research in radiation therapy service provision and reducing patient anxiety should focus on RT role definition, RT training in communication skills and detection and management of anxiety, referral pathways to psychosocial services and implementation of these processes into clinical practice.

## Conflict of Interest

The authors declare no conflict of interest.
